# Changes in out-of-home food purchasing following the introduction of England’s calorie labelling regulations: a population-level controlled interrupted time series analysis

**DOI:** 10.1136/bmjph-2025-003957

**Published:** 2026-04-27

**Authors:** Alexandra Irene Kalbus, Jean Adams, Kerry Ann Brown, Alan Brennan, Dalya Marks, Stephen O’Neill, Oana-Adelina Tanasache, Penny Breeze, Steven Cummins, Cherry Law, Richard Smith, Laura Cornelsen

**Affiliations:** 1 Population Health Innovation Lab, Department of Public Health, Environments and Society, Faculty of Public Health and Policy, London School of Hygiene & Tropical Medicine, London, UK; 2 MRC Epidemiology Unit, University of Cambridge, Cambridge, UK; 3 Department of Public Health and Sport Sciences, Faculty of Health & Life Sciences, University of Exeter, Exeter, UK; 4 Department of Population Health, Faculty of Epidemiology and Population Health, London School of Hygiene & Tropical Medicine, London, UK; 5 SCHARR, Sheffield Centre for Health and Related Research, Division of Population Health, School of Medicine and Population Health, University of Sheffield, Sheffield, UK; 6 Department of Public Health, Environments and Society, Faculty of Public Health and Policy, London School of Hygiene & Tropical Medicine, London, UK; 7 Department of Health Services Research and Policy, Faculty of Public Health and Policy, London School of Hygiene & Tropical Medicine, London, UK; 8 Department of Agri-Food Economics and Marketing, University of Reading, Reading, UK

**Keywords:** Public Health, Food Services, Program Evaluation

## Abstract

**Introduction:**

Large out-of-home (OOH) food businesses in England have been required by law to display calorie information on menus since 6 April 2022. This study investigated whether the implementation of this policy was associated with changes in calories purchased OOH by consumers.

**Methods:**

Controlled interrupted time series analysis was used to estimate changes in calories purchased from all OOH outlets in England (intervention group). Secondary outcomes included purchases from large chains, non-chain outlets and five subtypes of purchases (meals, lower-calorie coffee, higher-calorie coffee, sandwiches and fish and chip meals). The control series consisted of purchases from non-chain outlets in Scotland and Wales to avoid spillover labelling in chains across Great Britain. We aggregated self-reported itemised OOH food and non-alcoholic drink purchases from a rolling consumer panel of ~7500 individuals spanning 13 weeks pre intervention and 34 weeks post intervention to population-level average weekly per-person calorie (kcal) purchase estimates. Linear regression, adjusted for season and inflation, modelled level and trend changes compared with the counterfactual of no mandatory policy. Subgroup analyses explored effects by age, sex, occupational socioeconomic status, weight status and weekday/weekend purchases.

**Results:**

Compared with the counterfactual, we found no evidence of a change in overall calories purchased OOH associated with mandatory calorie labelling (level change −95.6 kcal, 95% CI −471.2 to 280.0; trend change 5.1 kcal, 95% CI −5.5 to 15.8). There was also no robust evidence of changes in calories purchased OOH for secondary outcomes and by subgroups compared with the counterfactual of no mandatory calorie labelling. Small changes observed in these analyses were sensitive to analytical choices.

**Conclusions:**

This study supports existing evidence that calorie labelling alone is unlikely to secure significant changes in food purchasing behaviour at population level. Possible changes to menus were not included in the study and warrant further investigation.

**WHAT IS ALREADY KNOWN ON THIS TOPIC**

- Large out-of-home (OOH) food businesses in England are required to show calories.
- Population-wide impact of the policy on consumer behaviour is unknown.

**WHAT THIS STUDY ADDS**

- Calorie labelling regulations were not associated with changes in calories purchased for OOH consumption at the population level.
- Exploratory analysis did not find changes in subgroups by sociodemographic characteristics.

**HOW THIS STUDY MIGHT AFFECT RESEARCH, PRACTICE OR POLICY**

- Information provision alone may not be enough to improve population-wide dietary health.

## Introduction

 Diet-related diseases are a global public health concern.^1 2^ More than 25% of UK adults live with obesity and a further 38% with overweight, with disadvantaged groups disproportionately affected.^3^ Diets high in energy-dense and nutrient-poor foods are a key contributor to unhealthy weight and poor dietary health. Food prepared away from home contributes on average around 300 kcal per person per day^4^ and tends to be less healthy compared with food prepared at home.^5 6^ Meals served in large UK restaurant and fast-food chains have been reported to typically exceed 600 kilocalories (kcal) recommended per serving.^7^,^9^

In an effort to improve population diets, mandatory calorie labelling in the out-of-home (OOH) food sector in England was introduced on 6 April 2022, following similar nation-wide and local policies in the USA, Canada, Australia and other countries.^10^ The calorie labelling regulations apply to all OOH food businesses with ≥250 employees that offer prepared food ready for immediate consumption.^11^ In 2022, businesses with ≥250 employees accounted for nearly half (47%) of the Accommodation and Food Service sector’s turnover.^12^ The calorie label must be displayed at the point of choice and in kcal, correspond to the serving size offered, and be accompanied by the statement ‘adults need around 2000 kcal a day’.^11^

Calorie labelling is hypothesised to reduce overall calories consumed, and thereby improve population diet, via two pathways. The first is via individual behaviour change whereby consumers select lower-calorie foods as a result of having access to nutritional information.^13^ This requires the consumer to notice this information, gain additional information through the label they would not have had otherwise and be able and willing to use it to make healthier food choices.^14^ The second mechanism is via change in the offer whereby mandating calorie labels to be shown encourages food businesses to change their menus by removing higher-calorie options, offer more lower-calorie options or reformulate existing dishes to reduce calorie content.^15^

Most evidence on the effectiveness of calorie labelling to improve diets focusses on individual behaviour change and originates from the USA, where despite a mixed evidence base, an overall significant calorie reduction following calorie labelling has been established by meta-analyses.^13^ The most recent meta-analysis suggests a reduction in selected calories of 11 kcal per meal.^16^

There is limited knowledge on the calorie labelling regulations’ impact on population health in England. Experimental studies that randomised participants into viewing menus with or without calories have found generally fewer calories ordered among those who were provided calorie labels.^17^,^19^ A modelling study assuming a 47 kcal-per-meal reduction suggested by a previous meta-analysis^20^ concluded that as currently implemented, the policy would reduce obesity prevalence by 0.31% over 20 years.^21^ Recently published studies conducted in real-world settings examining the effects of the mandatory policy have relied on surveys with limited geographical reach^22^ or have been limited to specific settings such as the workplace.^23^ To our best knowledge, there is no research to date considering the effects of the calorie labelling regulations on consumer behaviour at the population level in England.

In this study, we seek to address this knowledge gap by examining changes in OOH food and non-alcoholic drink purchasing following the introduction of mandatory calorie labelling in the OOH food sector in England using a controlled interrupted time series (CITS) design.

## Materials and methods

We adopted a CITS design to estimate the effect of England’s calorie labelling regulations on calories purchased from OOH food and non-alcoholic drinks using transaction-level consumer purchase data from January to November 2022. The CITS design, commonly used in public health research,^24^,^26^ compares observed purchases in England following implementation of calorie labelling on 6 April 2022 (the intervention) with a counterfactual where the policy had not been implemented. The inclusion of a control series allows one to estimate intervention effects in the presence of confounders that are common to both series, thereby avoiding bias due to temporal changes unrelated to the intervention.^27^

### Data

A detailed description of the data source and data preparation process is provided in online supplemental material 1. In brief, we used transaction-level food and drink purchasing data from Worldpanel by Numerator’s OOH Purchase Panel for the period 3 January 2022 to 27 November 2022. This rolling panel, consisting of approximately 7500 individuals annually who are representative in terms of individual characteristics (age group and sex) and region of residence of the population aged 13–79 years in Great Britain, continuously record purchases of prepared food and non-alcoholic drinks for consumption away from home or prepared food purchased for at-home consumption (takeaways), including via online food delivery. Individuals sign up to the consumer panel and receive rewards, for example, shopping vouchers, in return for recording their purchases. In 2022, individuals recorded purchases for an average of 16 weeks, with 54% of reporters recording in both pre-intervention and postintervention period.

Transaction-level purchase information was linked to a dataset provided by Worldpanel by Numerator of calorie information. This information was collected separately from retailer websites at one point in time, with most information collected between June and August 2022. Where no calorie information was available (68.90% of products), calorie values were imputed based on similar products purchased from similar stores (see online supplemental material 1).

Information on the individuals reporting OOH purchases included their age, sex, occupational socioeconomic status (SES), body mass index (BMI) calculated from self-reported height and weight, and region of residence. SES is based on the individual’s occupational social grade following the classification by the National Readership Survey.^28^ We operationalised SES as follows: high (AB: “Higher and intermediate managerial, administrative and professional” and C1: “Supervisory, clerical and junior managerial, administrative and professional”) and low (C2: “Skilled manual workers” and DE: “Semi-skilled and unskilled manual workers; and State pensioners, casual and lowest grade workers, unemployed with state benefits only”). SES was unknown for four individuals, who were excluded from the respective subgroup analysis. We used BMI information to determine the individual’s weight status as follows^29^: underweight and healthy weight, grouped together due to the low prevalence of underweight in the sample: <25 kg/m^2^; overweight: 25–29.9 kg/m^2^; obesity: ≥30 kg/m^2^. Due to the voluntary nature of BMI reporting, 26% of reporters’ weight status was unknown. Although missingness was not associated with calories purchased, findings of the subgroup analysis by weight status should be interpreted as exploratory. Region of residence was dichotomised into intervention (England, 88.9% of underlying reporters) and control group (Scotland and Wales, 7.1% and 4.0% of underlying reporters, respectively).

### Outcomes

The primary outcome was population-level mean calories (kcal) purchased from OOH food businesses per person per week. OOH food businesses were defined as any business that offered unpackaged, prepared food and drink ready for immediate consumption. Such businesses include restaurants, cafes, pubs and takeaways as well as all-paid-for workplace canteens and entertainment venues.

Secondary outcomes included calories purchased from large chain restaurants and takeaways, referred to as large chains. These were businesses that were sufficiently large (250+ employees) to be required to show calories. We identified 92 large chains from the data. We also considered purchases made from OOH food businesses excluding the identified chains, referred to as non-chains, to assess potential intervention effects beyond large chains. Further outcomes included purchases of calories from all meals (pre-defined by Worldpanel by Numerator in the purchase data), coffees (distinguishing higher-calorie and lower-calorie options defined as coffees with high milk content such as cappuccino and low milk content such as filter coffee), sandwiches (including baguettes, wraps and other types of bread, but excluding hot dogs and burgers) and fish and chip meals. These outcomes were chosen to reflect a range of products commonly purchased from chains for individual rather than shared consumption and were comparable across businesses, for example, precluding pizzas which are often for sharing, and burgers which may be sold with or without chips on the side.

### Analytical dataset

While mandated only in England, spillover effects of calorie labelling to Scotland and Wales were highly likely, as some large cross-border food businesses operate across the UK. Authors’ communication with The Food Foundation, an independent charity focused on improving food systems in the UK, and email correspondence with four large restaurant chains confirmed that these businesses implemented calorie labelling uniformly across their UK branches, effectively treating the regulations as UK-wide policy. These four businesses contributed 20.2% of OOH purchases in Scotland and Wales. In addition, emerging research suggests that calorie labelling was adopted widely across the UK by large chains.^30^ As this violates an identifying assumption, namely the stable unit treatment value assumption (SUTVA),^31^ we removed purchases from chains identified in the data from the control series. We are confident that we have captured the majority of large chains, as we estimate that sales from these chains account for 37.4% of OOH expenditure, comparable to 47% of turnover from businesses with ≥250 employees in the Accommodation and Food Service Sector.^12^

The study period was restricted to 3 January to 27 November 2022, comprising 13 weeks pre intervention and 34 weeks post intervention, excluding the Christmas period. The intervention was assumed to begin the week including the implementation date (6 April), which started on Monday, 4 April 2022. Data predating January 2022 were not sought due to this period coinciding with measures related to the COVID-19 pandemic, which restricted interaction with the OOH food sector.^32^ Study data included 542 671 purchase records (119 378 pre intervention and 423 293 post intervention) made during 331 966 purchase occasions (74 691 pre and 257 275 post).

Purchases of calories were then aggregated to weekly population-level purchases per person using sampling weights provided by Worldpanel by Numerator. The weights refer to the population purchasing food and drink away from home, consisting of ~50 million individuals aged 13–79 years in Great Britain, and were designed to achieve representativeness in terms of age, sex, presence of children in the household and region of residence. They also incorporate weighting specific to under-reporting of products and reporters’ SES. Further details of the weights are Worldpanel by Numerator proprietary information, but we confirmed that the weighting process was not changed during the study period, thus we have no reason to expect biases from applying weights (unpublished author communication). The final analytical dataset consisted of 47 weekly observations each in the intervention and control series.

### Covariates

To account for rapid rises in inflation throughout 2022, we included the monthly change rate in the UK Consumer Price Index (CPI) as a covariate.^33^ To account for seasonality of purchasing, we included indicator variables for spring, summer, autumn and winter expressed as 3-month periods.

### Statistical analysis

The primary analysis concerns calories purchased OOH overall, the primary outcome. We further assessed secondary outcomes and subgroups in an exploratory analysis, with the order of analyses shown in table 1.

**Table 1:** Order of analyses

Primary analysis	Analysis of the primary outcome (all calories purchased out of home per person per week)
Secondary analysis	Analysis of secondary outcomes: calories per person per week purchased from Large chains Non-chains Meals Coffees (higher-calorie and lower-calorie options) Sandwiches Fish and chip meals
Exploratory analysis	Subgroup analysis of the primary outcome by: Age group Sex Socioeconomic status Weight status Weekend/weekday purchase Takeaway/dine-in purchase
Robustness checks	Sensitivity analysis of primary and secondary outcomes: Including varying pre-intervention trends Purchases made for the individual themselves only Purchases made by individuals≥18 years only Excluding the top 1% and 10% of high-calorie purchase occasions Balanced observations Temporal falsification Including a price index

We modelled immediate (level) and trend changes in weekly per-person calories purchased OOH associated with the implementation of calorie labelling using linear regression.^34^ We explored non-linear trends by adding quadratic terms and found that these did not fit the data better than the linear models. Models for all studied outcomes were structured as follows (suppressing coefficients):

Estimated outcome=group + time + intervention + time after intervention + intervention × group + time after intervention × group + season + CPI change + CPI change × group.

Group is a binary indicator of whether the observation belongs to the intervention (1) or control series (0). Time (in weeks) was included as a linear term, which yielded a better model fit than a quadratic term. The intervention effect was modelled as level change (intervention × group) and trend change (time after intervention × group). Post-intervention trend (time after intervention) was set to 0 at the time of intervention and as 1, 2, …33 in the following weeks, facilitating interpretation of the intervention’s coefficient as level change. Inflation was allowed to have separate effects in the intervention and control series (CPI × group). Consequently, the counterfactual was constructed from a combination of pre-intervention purchasing levels in the intervention group, common pre-intervention trends and post-intervention level changes and purchasing trends in the control group.

At first, we fitted unadjusted and fully adjusted models allowing pre-intervention time trends to vary between treatment and control group, but found that the interaction term between group and time was non-significant, indicating parallel trends (see model building in online supplemental material 2: table S1). We therefore proceeded with the analysis without this interaction term. While there is some lack of consensus in the literature as to whether the inclusion of this term is necessary,^27 35^ we follow St.Clair et al.^36^ who argue that if there are parallel trends in the pre-intervention series, this interaction term should not be included to allow greater precision of the effect estimates.

We present level and trend changes for all analyses undertaken. Where residuals were found to be autocorrelated (using the Durbin-Watson test), we present robust standard errors. For the main outcome and where intervention effects were observed, we also present plots showing observed, predicted and counterfactual values.

### Subgroup analysis

In further exploratory analyses (see table 1), we explored effect heterogeneity in the primary outcome (weekly per-person calories purchased OOH) by available respondent characteristics, namely age (three age bands: <35 years; 35–54 years; 55+ years), sex (male, female), SES (high, low) and weight status (under and healthy weight, overweight, obesity). We also explored differences in whether purchases occurred as dine-in or takeaway, and whether they were made on a weekday or weekend. As these characteristics are generally associated with diet and dietary health,^37 38^ there is a need to understand whether the impact of calorie labelling varies among these groups. Data were separately aggregated to create subgroup datasets of weekly calories purchased in the intervention and control group. The intervention effect was estimated through the same model as described above using the stratified data. For the comparative analysis by weekday or weekend, the outcome was rescaled to calories purchased per person per day.

### Robustness checks

To understand the robustness of our findings to analytical choices made, we undertook several robustness checks (see table 1). First, we explored how sensitive findings were to our model specification by allowing the pre-intervention trends to vary between treatment and control group. Second, we restricted the purchase records to only those recorded as made for the individual, excluding purchases made for other adults or children. This reduced the transaction-level dataset to 57.8% of observations. Third, we restricted purchase records to those made by individuals aged 18 years and above as the policy is targeted at adults (98.7% of transactions). Fourth, to assess the influence of outlier observations, we excluded the top 1% and top 10% purchase occasions by calorie content, respectively. Fifth, we used balanced observations by restricting the post series to until 3 July 2022, leaving 13 weeks each pre intervention and post intervention. Sixth, we explored if the actual date of policy implementation (week 14, starting 4 April) reflected the start of the intervention by moving the start of the modelled intervention backwards by 1 month, to the week commencing on 7 March 2022. Some outlets may have introduced calorie labels on menus in anticipation of the policy ahead of 6 April. If the start of the intervention was correctly specified, we would expect to see smaller intervention effects by moving it backwards, particularly in the level change (as the true intervention was still captured in the post series). Lastly, to assess whether UK-wide CPI sufficiently captured the impact of inflation on OOH food and drink purchases, we included a price index of average price per item purchased OOH (considering items purchased from all OOH outlets) per week and group. This index was not sales weighted to avoid capturing possible substitution from more expensive to less expensive menu items that might have occurred as a response to inflation.

All data preparation and analysis tasks were carried out in R V.4.4.1, specifically using the packages tidyverse,^39^ performance,^40^ marginaleffects,^41^ report^42^ and rempsyc.^43^ Alpha, representing the type 1 error rate, was set as 0.05.

### Patient and public involvement

Due to the nature of this study, using secondary data in a prespecified quasiexperimental framework, it did not include patient and public involvement.

## Results

Table 2 shows descriptive statistics of population-level calories purchased in the intervention and control series. Per-person weekly average calories purchased OOH in the intervention series pre-intervention were 2348.6 kcal, of which 922.2 kcal were purchased from large chains. Overall, there was a decreasing trend in calories purchased OOH in both intervention and control groups, although this was not statistically significant for all outcomes studied. Note that the control series excludes purchases made from large chains and therefore levels are not directly comparable with the intervention series.

**Table 2:** Mean calories (kcal) per person per week purchased in intervention and control series before and after calorie labelling introduction

Outcome (kcal per person per week purchased OOH)	Mean (SD) kcal per person per week		
	Pre intervention (13 weeks)	Post intervention (34 weeks)	Δ pre intervention and post intervention
	Intervention series		
All calories purchased OOH	2348.6 (107.0)	2273.5 (140.4)	−3.2%
	Secondary outcomes		
From large chains*	922.2 (75.4)	911.7 (78.6)	−1.1%
From non-chains	1426.5 (71.8)	1361.8 (77.9)	−4.5%†
From meals	1548.9 (95.6)	1491.7 (113.1)	−3.7%
From higher-calorie coffees	98.0 (6.7)	94.3 (5.3)	−3.8%
From lower-calorie coffees	13.3 (1.1)	11.9 (0.9)	−10.5%†
From sandwiches	245.8 (21.4)	229.6 (18.1)	−6.6%†
From fish and chip meals	144.4 (13.9)	129.8 (16.5)	−10.1%†
	Control series		
All calories purchased OOH	1490.2 (153.9)	1489.1 (154.8)	−0.1%
	Secondary outcomes		
From large chains	Excluded		
From non-chains	1490.2 (153.9)	1489.1 (154.8)	−0.1%
From meals	1022.0 (114.4)	983.9 (132.5)	−3.7%
From higher-calorie coffees	59.2 (8.6)	56.5 (9.8)	−4.6%
From lower-calorie coffees	15.2 (2.1)	13.8 (2.3)	−9.2%
From sandwiches	159.4 (30.8)	166.5 (34.9)	+4.5%
From fish and chip meals	135.8 (26.2)	113.4 (29.1)	−16.5%†

Authors’ analysis of Worldpanel by Numerator’s OOH Purchase panel, 47 weeks ending 27 November 2022.

* Large chains denote restaurant and takeaway businesses identified in the data that have ≥250 employees; purchases from large chains were excluded from the control series because of cross-border spillover effects of labelling among businesses operating across the UK, therefore the absolute figures presented above are not directly comparable.

† p<0.05 (two-sample t-test, unequal variances).

OOH, out of home (excluding purchases from supermarkets).

### Main analysis

Effect estimates (level and trend coefficients) for all outcomes following the final model specification are shown in table 3. Full model coefficients and graphs are provided in online supplemental material 3.

**Table 3:** Immediate and trend effects of mandatory calorie labelling at population level

Outcome (OOH kcal per person per week)	Level change (95% CI)	% change (95% CI)*	P value	Trend change (95% CI)	% change (95% CI)*	P value
All calories purchased OOH	−95.6 (−471.2 to 280.0)	−4.1 (−20.1 to 11.9)	0.618	5.1 (−5.5 to 15.8)	0.2 (−0.2 to 0.8)	0.344
	Secondary outcomes					
From large chains†	−31.2 (−351.8 to 289.4)	−3.4 (−38.1 to 31.4)	0.849	9.2 (0.2 to 18.3)	1.0 (0.02 to 2.0)	0.046
From non-chains	−155.0 (−466.8 to 156.8)	−10.9 (32.7 to 11.0)	0.330	4.2 (−4.6 to 13.1)	0.3 (−0.3 to 0.9)	0.349
From meals	−78.6 (−390.0 to 232.9)	−5.1 (25.2 to 15.0)	0.621	8.3 (−0.6 to 17.1)	0.5 (−0.04 to 1.1)	0.067
From higher-calorie coffees	−6.3 (−28.0 to 15.3)	−6.4 (−28.6 to 15.6)	0.566	0.4 (−0.2 to 1.1)	0.4 (−0.2 to 1.1)	0.161
From lower-calorie coffees	1.2 (−2.8 to 5.1)	9.0 (−21.1 to 38.4)	0.565	0.3 (0.2 to 0.4)	2.3 (−1.5 to 3.0)	<0.001
From sandwiches	−31.7 (−106.4 to 43.0)	−12.9 (−43.3 to 17.5)	0.405	−2.0 (−4.1 to 0.2)	−0.8 (−1.7 to 0.1)	0.070
From fish and chip meals	13.2 (−53.3 to 79.7)	9.1 (−36.9 to 55.2)	0.697	0.1 (−1.8 to 2.0)	0.1 (−1.2 to 1.4)	0.893

Authors’ analysis of Worldpanel by Numerator’s OOH Purchase panel, 47weeks ending 27 November 2022.

* Refers to mean pre-intervention outcome levels in England.

† Large chains denote restaurant and takeaway businesses identified in the data that have ≥250 employees.

OOH, out of home (excluding purchases from supermarkets).

**Figure 1:**
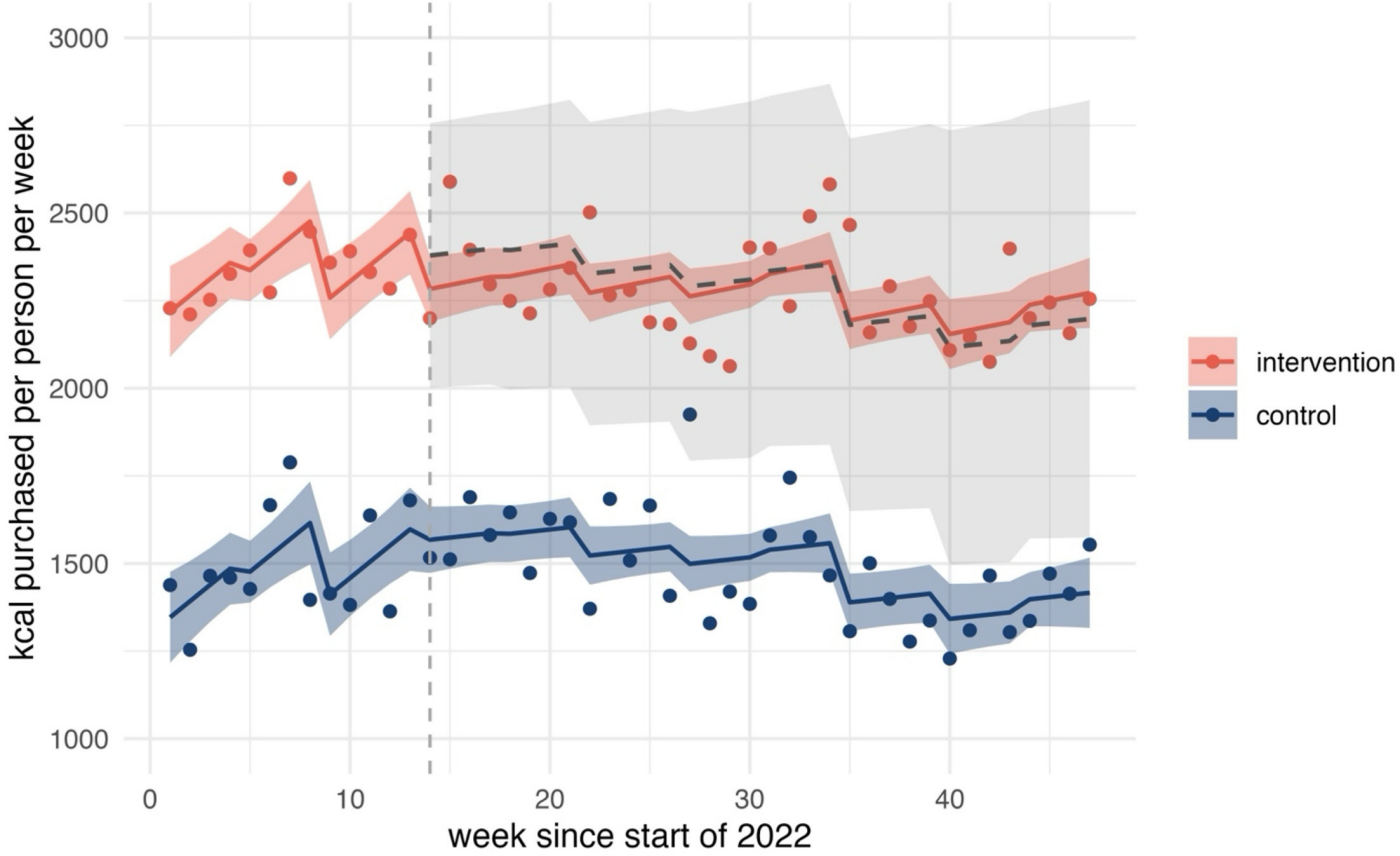
Overall calories purchased out of home. The graph shows observed (points) and predicted (solid lines) calories with counterfactual (grey dashed line) and 95% CIs (ribbons). Implementation of mandatory calorie labelling=week 14. The intervention series includes all out-of-home food and drink purchases in England. The control series is constructed from purchases made in Scotland and Wales and excludes purchases from large chains. Large chains denote restaurant and takeaway businesses identified in the data that have ≥250 employees. Authors’ analysis of Worldpanel by Numerator’s OOH Purchase panel, 47 weeks ending 27 November 2022.

**Figure 2:**
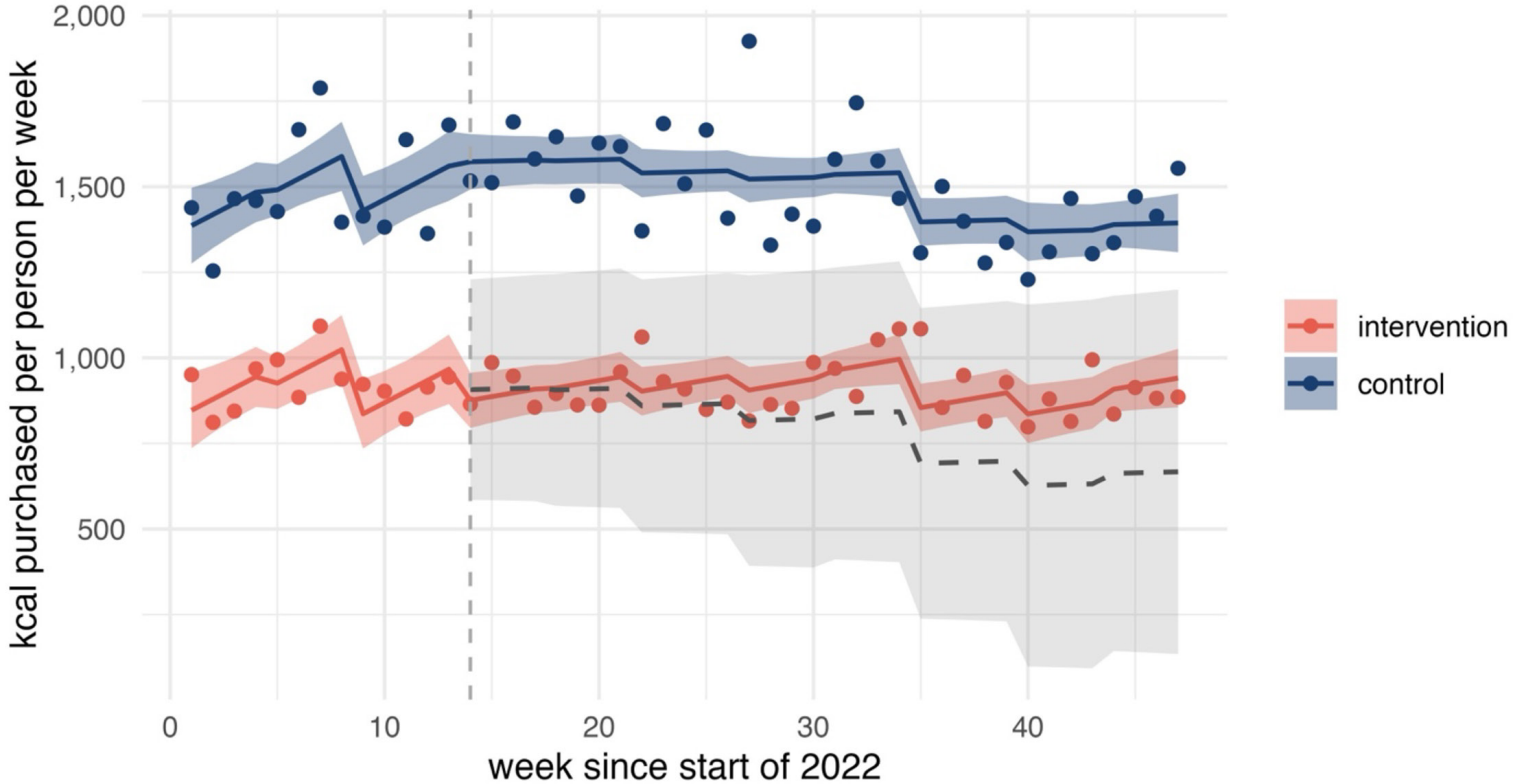
Calories purchased from large out-of-home chains. The graph shows observed (points) and predicted (solid lines) calories with counterfactual (grey dashed line) and 95% CIs (ribbons). Implementation of mandatory calorie labelling=week 14. Large chains denote restaurant and takeaway businesses identified in the data that have ≥250 employees. The intervention series includes purchases made from large out-of-home chains in England. The control series is constructed from purchases made in Scotland and Wales and excludes purchases from large chains. Authors’ analysis of Worldpanel by Numerator’s OOH Purchase panel, 47 weeks ending 27 November 2022.

**Figure 3:**
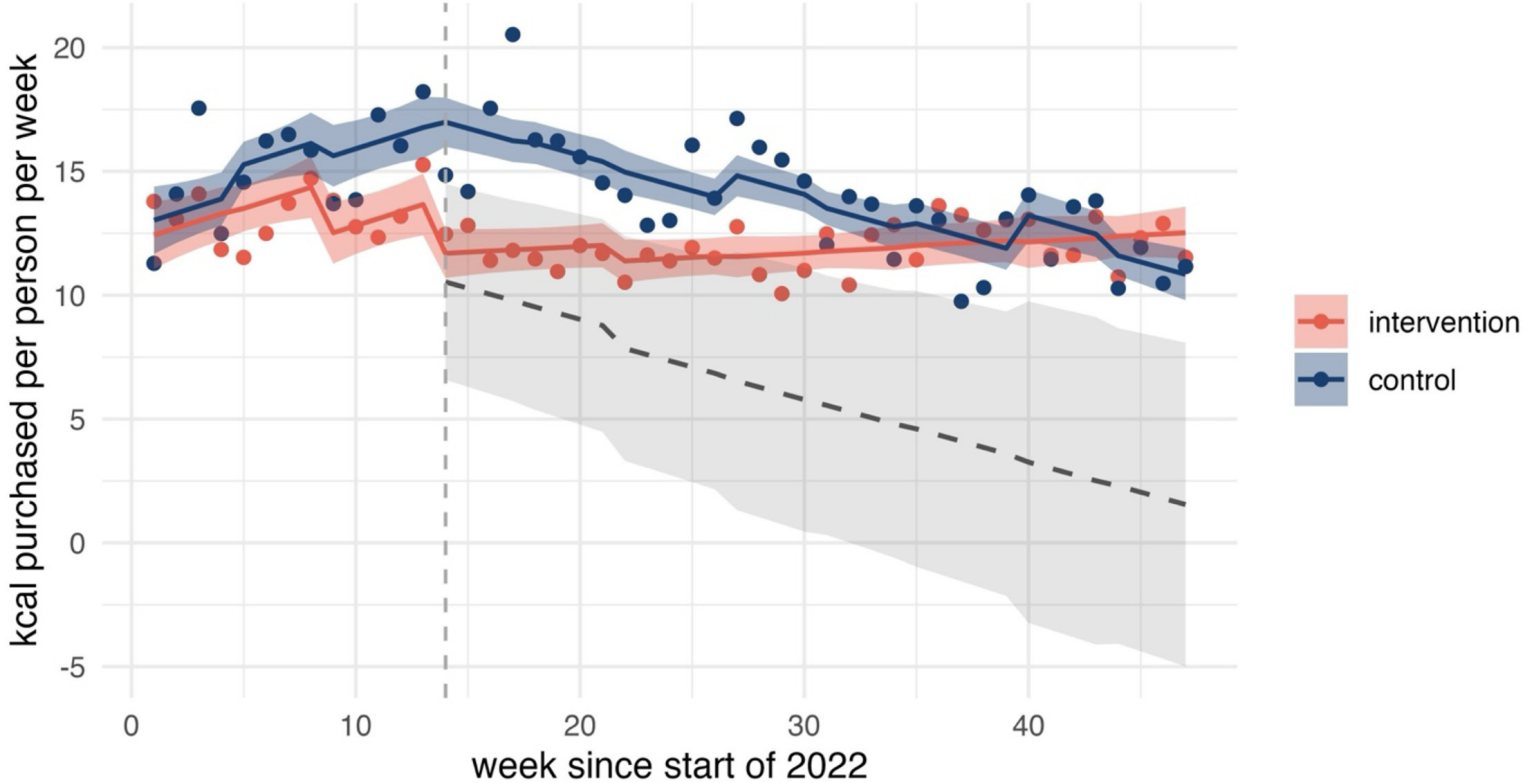
Calories purchased from lower-calorie coffees. The graph shows observed (points) and predicted (solid lines) calories counterfactual (grey dashed line) 95% CIs (ribbons). Implementation of mandatory calorie labelling=week 14. The intervention series includes purchases in England. The control series is constructed from purchases made in Scotland and Wales and excludes purchases from large chains. Large chains denote restaurant and takeaway businesses identified in the data that have ≥250 employees. Authors’ analysis of Worldpanel by Numerator’s OOH Purchase panel, 47 weeks ending 27 November 2022.

All of the following changes are with respect to the counterfactual where mandatory calorie labelling had not been implemented. We observed no evidence of changes in calories purchased OOH. Observed, predicted and counterfactual values for calories purchased OOH are shown in figure 1. There was some evidence of an increasing trend in calories purchased from chains by 9 kcal per person per week (95% CI 0.15 to 18.32) (see figure 2). Further, there was strong evidence of an increasing trend in calories purchased from lower-calorie coffees (0.3 kcal per person per week, 95% CI 0.19 to 0.41) (see figure 3). We did not find statistically significant changes at conventional levels in calories purchased OOH in any of the remaining outcomes either as immediate (level) or trend changes associated with mandatory calorie labelling.

### Subgroup analysis

Results from the subgroup analysis are provided in online supplemental material 4. We observed no evidence of effects of mandatory calorie labelling on calories purchased OOH in the analysed subgroups by age, sex, SES and whether it was a dine-in/takeaway or weekday/weekend purchase. However, we found some evidence for an increasing trend in calories purchased OOH of 14 kcal per person per week (95% CI 0.4 to 28.3) associated with calorie labelling among people living with overweight.

### Robustness checks

Online supplemental material 5 contains the robustness checks undertaken. When allowing pre-intervention trends to vary by group, point estimates were broadly in line with our main analysis, while CIs were wider, resulting in no statistically significant intervention effects. Similarly, no intervention effects were observed when using balanced observations as well as including purchases from individuals ≥18 years only. Findings from an analysis that considered only purchases made for the individual themselves were similar to the main analysis with the following exceptions: the trend change in calories purchased from large chains, which was no longer statistically significant, while there was an increase in calories from OOH meals (3.7 kcal per person per week, 95% CI 0.01 to 7.5) and higher-calorie coffees (0.47 kcal per person per week, 95% CI 0.06 to 0.89) but a decrease of calories purchased from sandwiches (−2.5 kcal per person per week, 95% CI −4.3 to −0.64). Findings were similar to those observed in the main analysis when excluding the top 1% of occasions by calorie content, with the exception that we did not observe the trend change in calories purchased from large chains. Excluding the top 10% of purchase occasions by calorie content also led to similar results for the main outcome and lower-calorie coffee, while the increasing trend change for calories purchased from large chains was not observed. However, this analysis indicated an immediate (level) reduction of 97 kcal per person (95% CI −188.8 to −5.2) from OOH meals, and a negative trend change in calories purchased from fish and chip meals (−1.3 kcal per person per week, 95%CI −2.0 to −0.6). When considering the beginning of the intervention period on 7 March 2022, we observed no intervention effects except the positive trend change in calories from lower-calorie coffee. Finally, including a price index did not change results.

## Discussion

### Summary of findings

This study aimed to evaluate the impact of implementing mandatory calorie labelling in large food businesses in England on population-level OOH food and drink purchasing. Using transaction-level purchase data in a CITS design, we found limited robust evidence of changes in consumer purchasing following implementation of the calorie labelling regulations. While there was no evidence for an intervention effect on overall calories purchased OOH, the primary outcome, we observed changes among secondary outcomes, specifically increases in calories purchased following policy implementation from large chains as well as from lower-energy coffee compared with the counterfactual. However, these findings were sensitive to the analytical specifications tested in robustness checks and should thus be interpreted with caution. Exploratory subgroup analyses were mostly in agreement with the main finding of no changes in overall calories purchased OOH, except for an increasing trend in purchases among individuals living with overweight.

### Comparison with other studies

The evidence on the effectiveness of calorie labelling on menus remains mixed. A recent Cochrane systematic review and meta-analysis reported a reduction of 1.8% in calories selected, corresponding to 11 kcal of a typical meal, as a result of calorie labelling.^16^ However, the studies that informed this estimate were predominantly conducted in laboratory settings in the USA and included different types of calorie labels. In contrast, an earlier review of high-quality real-world studies with the majority employing text-based labels akin to those mandated by the English calorie labelling regulations found no change in calories selected.^44^ Findings from these reviews may not be directly applicable to England because of differences in food environments and how calorie labels are presented in terms of text/visual label and business compliance.

In England, a natural experiment assessing purchases from workplace canteens reported no change in calories purchased, but found small reductions in the mean calorie content of menu options at each menu change.^23^ Another study using restaurant customer intercept surveys in four local areas before and after implementation of mandatory calorie labelling reported an increase in noticing calorie labels but found no changes in calories purchased or consumed.^22^ Our study, finding no evidence of an impact of mandatory calorie labelling on calories purchased OOH, aligns with this emerging evidence base.

Findings from our subgroup analysis have to be interpreted with caution as Worldpanel by Numerator sample weights are not constructed to reflect this subgroup split and weight status was not available for 26% of underlying reporters due to the voluntary nature of BMI reporting. Nevertheless, our findings for age and SES are broadly in line with previous research which has not found effect variation by sociodemographic characteristics. A pooled analysis of 12 randomised controlled trials, of which 5 were UK-based, showed no effect modification by participants’ age and SES,^45^ while a real-world customer intercept study in England did not find varying effects by age, gender, ethnicity or SES.^22^ Differential impacts of calorie labelling may be more related to individual value orientation and less to sociodemographic characteristics.^46 47^

### Interpretation of findings

The present study adds to the emerging evidence base finding no impact of mandatory calorie labelling on menus alone on consumer behaviour change in England. Among secondary outcomes, we did observe some evidence, paradoxically, for an increasing trend in calories purchased from large OOH chains following the implementation of mandatory calorie labelling. This finding, however, needs to be interpreted with caution as it was not robust to analytical choices. While the increasing trend in lower-calorie coffees is seemingly in line with our hypothesis of substitution away from higher-calorie coffees, it was not robust to the model specification of varying pre-intervention trends by group. We also observed an increasing trend in calories purchased OOH among individuals with overweight. However, this exploratory finding is supported only by moderate evidence (p=0.044) and constructed from underlying data with a high rate of missing values (26%). Further research should specifically examine the relationship between mandatory calorie labelling, behaviour change and weight status in England.

Though not a primary objective of the study, we observed decreases in calories purchased OOH in both intervention and control groups following on from a steep increase in purchasing at the beginning of 2022. This initial increase may be explained by the start of the year coinciding with the end of various COVID-19 pandemic-related restrictions affecting social and public life as well as individuals’ engagement with the OOH food sector.^32^ The following decrease may be a stabilising of behaviour and/or a response to inflation, which spiralled in spring 2022.^48^

We may have underestimated the effect of mandatory calorie labelling in our study due to study design elements. Specifically, the policy was not a sharp implementation as 21% of large food businesses had already provided calorie labels in the year prior to the policy, although inconsistently,^49^ including in the control group.^50^ Click or tap here to enter text. As such, we estimated the effect of mandatory calorie labelling compared with no and voluntary labelling. With respect to possible anticipatory effects, we concluded from our robustness check of ‘moving’ the intervention to a month earlier (7 March) which observed no effects that bias from anticipatory effects is unlikely. We were not able to explore setting the intervention date earlier, as that would have coincided with pandemic-related restrictions affecting the OOH food sector. We further estimated an intention-to-treat effect, as not every individual in the intervention group was exposed to calorie labelling due to business eligibility, compliance or individuals noticing calorie labels, as discussed in more detail below. Finally, our analysis did not capture possible reformulation or menu changes. Previous research suggests an average reduction of 9 kcal (95% CI −16 to −1) per menu item among large UK businesses following the calorie labelling regulations.^51^ The present study offers an evaluation of the policy’s impact on population-wide consumer behaviour, while the combined effect of consumer behaviour change and menu change has been examined based on previous systematic reviews.^21^

Yet, our study’s findings are in line with the emerging literature on the calorie labelling regulations’ impact in England. A reason for the calorie labelling regulations’ lack of impact may be its reliance on behaviour change. The high level of individual agency required to benefit from calorie labelling, namely to understand and use calorie labels as a guide to healthier eating as well as a drive and willingness to change behaviour, may explain the policy’s limited effectiveness.^52^ Businesses commonly favour interventions focussing on individual behaviour change, including information provision, awareness raising and communication with consumers, despite these being less effective than structural changes involving pricing, reformulation and advertising restrictions.^53^

Another explanation, particularly pertaining to English context, may be the extent of exposure to calorie labelling dependent on (1) how many businesses are eligible for calorie labelling; (2) business compliance with the regulations; and (3) customers noticing labels. While outlets under the legislation scope represented nearly half (47%) of the Accommodation and Food Service Sector’s turnover in 2022,^12^ a fifth did not provide calorie information at all and only 15% met all guidelines of showing calories, with non-compliance particularly in relation to presenting information clearly and prominently.^54^ Additionally, even if calorie labels are present, these go widely unnoticed by consumers. Previous research recorded only 23% of takeaway consumers noticing calorie labels in past online orders, and of those, only 26% using them to reduce calories purchased.^55^ Consequently, if calorie labelling were effective in reducing calories purchased, it is likely that its effect would have been diluted as far as to not be detectable at population level.

The limited evidence of the calorie labelling regulations’ effectiveness on reducing overall calorie intake is juxtaposed by the opposition to the policy by people with lived experience of eating disorders due to concerns of relapsing into more severe periods of disordered eating.^56^ Qualitative research among people with experience of eating disorders in England also suggests that calorie labelling adversely impacts eating disorders and/or recovery.^57^ In this context, the risks posed to people with eating disorders need to be weighed against the efficacy of calorie labelling identified.

### Limitations

While CITS is a strong design for inferring causality,^58^ the following limitations pertain to our study. First, the key identifying assumption of no interference, or SUTVA,^31^ may have been violated. Although calorie labelling is only mandatory in England, several large cross-border chains have introduced calorie labels uniformly across Great Britain.^30^ We reduced the bias from such spillover effects by removing purchases from identified large businesses from the control series, yet we cannot rule out that some outlets showing calories remained in the control series. Consequently, our analysis may have underestimated intervention effects. As our analysis has captured most large, nation-wide chains and identified an estimated 79% of sales from large chains, we believe that the extent of this bias is limited. Second, the common shock assumption, which requires idiosyncratic shocks in the post-intervention period to be similar in treatment and control group,^59^ may have been violated as the calorie labelling regulations’ implementation coincided with a period of high inflation^60^ which could have impacted the frequency, level and types of OOH purchases differentially. As only UK-wide inflation data are available, we had to assume that inflation rates were the same in intervention (England) and control series (Scotland and Wales). To mitigate this, we allowed the effects of inflation on calories purchased to vary by group. Findings were robust to including a further, group-specific price index. We are not aware of other events impacting OOH purchasing in intervention and control series differently. Fourth, the brief pre-intervention period (13 weeks) may preclude the assessment of seasonal patterns and the detection of small effects due to reduced statistical power. The pre-intervention period was restricted to the start of the year, coinciding with the end of COVID-19-related restrictions on hospitality, which differed across Great Britain. Fifth, panellists select into the consumer panel, therefore we cannot fully rule out selection bias despite demographic weighting. Finally, calorie information was less available for purchases from non-chains, which is particularly relevant for the control series.

## Conclusion

This study used a natural experiment design to assess the impact of the calorie labelling regulations in England on calories purchased from OOH food venues. No robust evidence of changes in purchasing of OOH foods and non-alcoholic drinks related to the policy was observed, which is in line with existing evidence that calorie labelling alone is unlikely to secure significant changes in food purchasing behaviour. Further research may ascertain if the policy led to a reduction in calories offered by restaurants and thereby led to changes in overall calorie consumption.

## Supplementary material

10.1136/bmjph-2025-003957 online supplemental file 1

10.1136/bmjph-2025-003957 online supplemental file 2

10.1136/bmjph-2025-003957 online supplemental file 3

10.1136/bmjph-2025-003957 online supplemental file 4

10.1136/bmjph-2025-003957 online supplemental file 5

## Data Availability

Data may be obtained from a third party and are not publicly available.
