# Supplementary material for: Changes in out-of-home food purchasing following the introduction of England’s calorie labelling regulations: a population-level controlled interrupted time series analysis
Source: BMJ Public Health. 2026 Apr 27;4(2):e003957. doi: 10.1136/bmjph-2025-003957 (PMC13141202; doi:10.1136/bmjph-2025-003957)
Supplement: online supplemental file 1 [file bmjph-4-2-s001.pdf]

Changes in out-of-home food purchasing following the introduction of England's calorie labelling regulations: a population-level controlled interrupted time series analysis

Supplementary Material 1 – Description of data cleaning process

Description of data cleaning process

We used transaction-level food and drink purchasing data from Worldpanel by Numerator's Out-of-Home Purchase Panel for the period 3rd January 2022 to 27th November 2022. This contains information on purchases of prepared food and non-alcoholic drinks for consumption away from home or prepared food purchased for at-home consumption (takeaways). Purchases are continuously recorded by a sample of ~7,500 individuals that are part of Worldpanel by Numerator's Take-home panel of ~30,000 households across Great Britain who record all take-home purchases. The sample of out-of-home (OOH) purchasers are representative in terms of individual characteristics (age group and sex) and region of residence of the population aged 13–79 years in Great Britain.

Panellists record OOH purchases via a mobile phone application. The dataset available for this research included the respective food and drink item's name, product identifier, and price; the date of purchase; an identifier of the purchase occasion, e.g. where several items were bought together; where it was bought from (store identifier), which includes names of larger retailers and generic categories for smaller businesses, e.g. 'café'; and who it was bought for, e.g. for the individual themselves or other adults or children.

Worldpanel by Numerator provided a dataset of calorie information separately collected from retailer websites. These data were available for one point in time, with most information collected between June and August 2022. For products for which no calorie information was available (68.90% of products), predominantly purchased from small businesses not required to display this information, or products that are on the menus temporarily and are not eligible for labelling regulations, imputed calorie information was provided. These imputed calories were calculated as trimmed means based on similar items. This matching process systematically followed a hierarchy specific to the type of product, starting at similar items in similar stores within similar channels down to matching on similar stores. The majority of imputed values were based on similar items such as sandwiches with the same filling or meals with the same protein (91%), while 41% were based on such similar items within the same or similar stores. For instance, calories for a cheese sandwich sold in a café would have been imputed using calorie information from other sandwiches in the same outlet, if available, or based on cheese sandwiches from other cafés. We linked calorie information and purchase records by product and outlet (97.32% of purchase records), and non-outlet specific products (such as branded bottled drinks) by product identifier only (2.08% of purchase records). For the remaining unmatched purchase records, nutrition information was retrieved from Worldpanel by Numerator Take-home Panel Data if the products were barcoded, e.g. bottled drinks and packaged snacks (0.07%), looked up manually on businesses' websites (0.04%) or imputed based on similar products in similar types of outlets (0.50%).

Outcomes

The primary outcome was population-level mean calories (kcal) purchased from OOH food businesses per person per week. OOH food businesses were defined as any

business that offered unpackaged, prepared food and drink ready to be consumed, irrespective of its size. Such businesses include restaurants, cafes, pubs, and takeaways as well as workplace canteens and entertainment venues. Purchases from supermarkets and other retail outlets as well as school canteens that were not targeted by the regulations were excluded.

Secondary outcomes included population-level calories per person per week from large chain restaurants and takeaways for which names were included in the dataset (as these are preset in the mobile application where purchases are recorded), referred to hereafter as large chains. These were businesses we could identify by name in the data and were sufficiently large (250+ employees) to be required to show calories. However, they do not include the totality of large OOH food businesses in England, as not all large chains are named in the data. We identified 92 large chains from the data which represented approximately one fifth of all large businesses (250+ employees) in the Accommodation and Food Service sector [1]. We estimate that these chains account for 37.4% of expenditure based on the purchase data, compared to 47% of turnover from businesses with ≥ 250 employees in the Accommodation and Food Service Sector [1]. We also considered purchases made from OOH food businesses excluding the identified chains, referred to as non-chains. This outcome was chosen to assess the effects of voluntary labelling in smaller businesses from possible substitution from restaurants with calorie labelling to those without (albeit we cannot rule out that some purchases in this group were from large businesses that were not named and therefore not identified as such). Further outcomes included purchases of calories from all meals (pre-defined by Worldpanel by Numerator in the purchase data), coffees (distinguishing higher- and lower-calorie options defined as coffees with high milk content such as cappuccino, mocha, latte, and low milk content such as americano, filter coffee, macchiato, cortado, respectively), sandwiches (including baguettes, wraps and other types of bread, but excluding hot dogs and burgers), and fish and chip meals. These outcomes were chosen to reflect a range of products commonly purchased from chains for individual rather than shared consumption and were comparable across businesses, e.g. precluding pizzas which are often for sharing, and burgers which may be sold with or without chips on the side. While both Worldpanel by Numerator's Take-home Panel [2,3] and OOH Purchase Panel [4] data have been used extensively in public health research, this study is, to the best of our knowledge, the first to use the nutritional data compiled for the OOH Panel. Our estimate of calories purchased in OOH settings (336 kcal per person per day) is similar to a 2021 estimate of average population-level OOH purchases of 300 kcal per person per day (see Table 1 and [5]).

References

1. Office for National Statistics. Non-financial business economy, UK: employment size-band. 2025 [cited 13 Aug 2025]. Available: https://www.ons.gov.uk/businessindustryandtrade/business/businessservices/datasets/uknonfinancialbusinesseconomyannualbusinesssurveyemploymentsizeband/current
2. Yau A, Berger N, Law C, Cornelsen L, Greener R, Adams J, et al. Changes in household food and drink purchases following restrictions on the advertisement of high fat, salt, and sugar products across the Transport for London network: A controlled interrupted time series analysis. PLoS Med. 2022;19: e1003915. doi:10.1371/journal.pmed.1003915
3. Anderson P, O'Donnell A, Llopis EJ, Kaner E. The COVID-19 alcohol paradox: British household purchases during 2020 compared with 2015-2019. PLoS One. 2022;17: e0261609. doi:10.1371/JOURNAL.PONE.0261609
4. Law C, Smith R, Cornelsen L. Place matters: Out-of-home demand for food and beverages in Great Britain. Food Policy. 2022;107: 102215. doi:10.1016/j.foodpol.2021.102215
5. Mariani E, Chacko A, Stewart I, Hadley M, Sleeman C, Bowes Byatt L, et al. How eating out contributes to our diets. 2024. Available: https://www.nesta.org.uk/report/how-eating-out-contributes-to-our-diets/
