# Supplementary material for: Changes in out-of-home food purchasing following the introduction of England’s calorie labelling regulations: a population-level controlled interrupted time series analysis
Source: BMJ Public Health. 2026 Apr 27;4(2):e003957. doi: 10.1136/bmjph-2025-003957 (PMC13141202; doi:10.1136/bmjph-2025-003957)
Supplement: online supplemental file 2 [file bmjph-4-2-s002.pdf]

Changes in out-of-home food purchasing following the introduction of England's calorie labelling regulations: a population-level controlled interrupted time series analysis

Supplementary Material 2 – Model building

Model Building

Table S1. Model coefficients (95% confidence interval) for different specifications predicting all calories purchased OOH

	**Unadjusted model with varying pre-intervention trends**		**Fully adjusted model with varying pre-intervention trends**		**Final model – fully adjusted without varying pre-intervention trends**	
	Coefficient (95% confidence interval)	P	Coefficient (95% confidence interval)	P	Coefficient (95% confidence interval)	P
Intervention x group (level change)	-143.4 (-402.0 to 115.1)	0.273	-67.8 (-477.1 to 341.5)	0.743	-95.6 (-476.9 to 285.7)	0.619
Time after intervention x group (trend change)	3.6 (-25.5 to 32.6)	0.808	-1.2 (-35.9 to 33.4)	0.943	5.1 (-5.7 to 16.0)	0.347
Group	855.7 (655.6 to 1,055.8)	**<0.001**	1,133.3 (-79.5 to 2,346.0)	0.067	968.3 (108.7 to 1,827.9)	**0.028**
Time	11.9 (-8.2 to 31.9)	0.242	42.0 (6.1 to 77.9)	**0.022**	46.3 (18.2 to 74.4)	**0.002**
Time x group	0.45 (-27.8 to 28.7)	0.975	8.6 (-35.8 to 53.0)	0.701		
Intervention	39.9 (-143.0 to 222.6)	0.666	66.3 (-235.0 to 367.5)	0.663	80.2 (-210.8 to 371.1)	0.585
Time after intervention	-19.4 (-39.9 to 1.12)	0.065	-36.9 (-69.4 to -4.5)	**0.026**	-40.1 (-67.9 to -12.3)	**0.005**
Season (spring)			-187.1 (-388.5 to 14.3)	0.068	-187.1 (-387.4 to 13.2)	0.067
Season (summer)			-250.4 (-490.5 to -10.3)	**0.041**	-250.4 (-489.2 to -11.6)	**0.040**
Season (autumn)			-409.7 (-717.4 to -101.9)	**0.010**	-409.7 (-715.7 to -103.6)	**0.009**
CPI change			-60.9 (-222.3 to 100.6)	0.456	-78.1 (-212.2 to 56.10)	0.250
CPI change x group			-51.9 (-275.8 to 172.0)	0.646	-17.5 (-153.1 to 118.2)	0.799
Constant	1,419.1 (1277.7 to 1560.6)	**<0.001**	1,693.2 (807.0 to 2579.4)	**<0.001**	1,775.7 (1,002.5 to 2,549.0)	**<0.001**
Adjusted R^2	0.90		0.91		0.91	
BIC	1,222.6		1,230.9		1,226.5	

CPI = consumer price index; OOH = out of home. Authors' analysis of Worldpanel by Numerator's OOH Purchase panel, 47w/e, 27th Nov 2022.
