# Supplementary material for: Changes in out-of-home food purchasing following the introduction of England’s calorie labelling regulations: a population-level controlled interrupted time series analysis
Source: BMJ Public Health. 2026 Apr 27;4(2):e003957. doi: 10.1136/bmjph-2025-003957 (PMC13141202; doi:10.1136/bmjph-2025-003957)
Supplement: online supplemental file 4 [file bmjph-4-2-s004.pdf]

Changes in out-of-home food purchasing following the introduction of England's calorie labelling regulations: a population-level controlled interrupted time series analysis

Supplementary Material 4 – Subgroup analysis

Description of calories purchased out of home by subgroup

Table S2. Unadjusted kcal purchased OOH per person and week by subgroups

Subgroup	Average kcal purchased from OOH venues per person per week (SD)							
	Intervention series				Control series			
Age	% underlying reporters	Pre intervention	Post intervention	Δ pre- and post-intervention	% underlying reporters	Pre intervention	Post intervention	Δ pre- and post-intervention
< 35 years	19%	2,239.3 (219.2)	2,008.37 (186.3)	-230.9*	13%	865.5 (320.9)	856.0 (244.9)	-9.4
35–54 years	48%	2,335.3 (187.2)	2,296.6 (109.2)	-38.7	46%	1,255.9 (230.6)	1,361.1 (188.0)	105.2
55+ years	34%	2,456.5 (113.8)	2,493.1 (132.1)	36.6	40%	1.964.8 (141.6)	1,949.0 (204.1)	-15.7
Sex								
Women	63%	2,300.8 (137.7)	2,265.0 (151.7)	-35.9	60%	1,481.7 (240.4)	1,457.3 (182.5)	-24.4
Men	38%	2,396.9 (110.1)	2.282.0 (145.3)	-114.9*	40%	1,498.3 (139.1)	1,519.0 (232.1)	20.7
SES								
High	65%	2,381.2 (116.7)	2,278.9 (165.5)	-102.3*	62%	1,516.7 (192.4)	1,561.4 (166.3)	44.8
Low	35%	2,292.3 (153.9)	2,265.0 (147.4)	-27.3	37%	1,456.7 (218.0)	1,396.4 (244.5)	-60.4
Weight status								
Under and healthy weight	29%	2,415.2 (173.4)	2,255.1 (157.6)	-160.1*	26%	1,355.2 (162.9)	1,354.1 (240.6)	-1.1
Overweight	24%	2,153.9 (143.2)	2,180.8 (168.6)	26.9	27%	1,433.0 (142.2)	1,445.1 (229.7)	12.1
Obesity	21%	2,786.3 (187.8)	2,696.5 (221.4)	-89.7	24%	1,661.6 (327.3)	1,688.7 (293.6)	27.1
Takeaway vs dine-in								
Takeaway	87.5%	4,009.4 (249.5)	3,862.7 (235.2)	-146.7	79.6%	2,091.3 (393.9)	2,122.6 (311.7)	31.3
Dine-in	87.9%	1,626.2 (107.5)	1,687.5 (140.4)	61.3	86.0%	1,206.7 (178.5)	1,246.9 (151.2)	40.2
	Average kcal purchased from OOH venues per person per day (SD)							
Day of the week		Pre intervention	Post intervention			Pre intervention	Post intervention	
Weekday	88%	351.3 (27.9)	346.5 (26.8)	-4.8	84%	234.3 (28.1)	230.9 (28.1)	-3.4
Weekend	90%	865.4 (59.1)	814.8 (43.5)	-50.5*	88%	526.9 (62.7)	519.0 (74.6)	-7.9

OOH = out of home; SES = socio-economic status. The intervention series includes all out of home (OOH) purchases in England. The control series is constructed from purchases made in Scotland and Wales and excludes purchases from large chains. Large chains denote restaurant and takeaway businesses identified in the data that have ≥250 employees. Authors' analysis of Worldpanel by Numerator's OOH Purchase panel, 47w/e, 27th Nov 2022. OOH = out-of-home. * $p<0.05$ (two-sample t-test, unequal variances).

Changes in calories purchased OOH following England's calorie labelling regulations by subgroup

Table S3. Immediate and trend effects of mandatory calorie labelling on calories purchased from OOH venues per person and week by subgroup

Subgroup	Average kcal purchased from OOH venues per person per week (95%CI)					
Age	Level change (95%CI)	% change (95%CI)[a]	P	Trend change (95%CI)	% change (95%CI)[a]	P
< 35 years	-555.3 (-1215.1 to 104.6)	-22.6 (-49.5 to 4.3)	0.099	-2.4 (-21.1 to 16.4)	-0.1 (-0.9 to 0.7)	0.806
35–54 years	178.2 (-399.9 to 756.3)	7.7 (-17.1 to 32.4)	0.546	7.6 (-8.8 to 23.8)	0.3 (-0.4 to 1.0)	0.364
55+ years	21.5 (-402.5 to 445.5)	0.9 (-16.4 to 18.1)	0.921	2.8 (-9.2 to 14.8)	0.1 (-0.4 to 0.6)	0.651
Sex						
Women	-199.1 (-695.3 to 297.1)	-8.7 (-30.2 to 12.9)	0.432	3.7 (-10.4 to 17.8)	0.2 (-0.5 to 0.8)	0.605
Men	-0.31 (-470.0 to 469.3)	-0.0 (-19.6 to 19.6)	0.999	6.9 (-6.3 to 20.0)	0.3 (-0.3 to 0.8)	0.305
SES						
High	-136.2 (-588.6 to 316.1)	-5.7 (-24.7 to 13.3)	0.555	2.3 (-10.5 to 15.2)	0.1 (-0.4 to 0.6)	0.721
Low	-32.1 (-569.1 to 505.0)	-1.4 (-24.8 to 22.0)	0.907	7.7 (-7.5 to 23.0)	-0.3 (-0.3 to 1.0)	0.321
Weight status						
Under and healthy weight	-357.3 (-867.4 to 152.7)	-14.8 (-35.9 to 6.3)	0.170	-1.7 (-16.2 to 12.8)	-0.1 (-0.7 to 0.5)	0.821
Overweight	-31.6 (-524.0 to 460.8)	-1.5 (-24.3 to 21.4)	0.900	**14.3 (0.4 to 28.3)**	**0.7 (0.02 to 1.3)**	**0.044**
Obesity	411.4 (-347.4 to 1170.2)	14.8 (-12.5 to 42.0)	0.288	8.3 (-13.2 to 29.8)	0.3 (-0.5 to 1.1)	0.449
Takeaway vs dine-in						
Takeaway	-91.8 (-485.1 to 301.4)	-2.3 (-12.1 to 7.5)	0.647	3.7 (-7.5 to 14.9)	-0.1 (-0.2 to 0.4)	0.515
Dine-in	121.0 (-678.3 to 920.3)	7.4 (-41.7 to 56.6)	0.767	16.9 (-5.8 to 39.5)	-1.0 (-0.4 to 2.4)	0.145
	Average kcal purchased from OOH venues per person per day (95%CI)					
Day of the week	Level change (95%CI)		P	Trend change (95%CI)		P
Weekday	-24.0 (-95.5 to 47.4)	-6.8 (-27.2 to 13.5)	0.510	0.24 (-1.8 to 2.3)	0.1 (-0.5 to 0.7)	0.814
Weekend	1.5 (-172.1 to 175.1)	0.2 (-19.9 to 20.2)	0.987	3.0 (-2.0 to 7.9)	0.3 (-0.2 to 0.9)	0.239

95%CI = 95% confidence interval; OOH = out of home (excluding purchases from supermarkets); SES = socio-economic status. [a] Refers to mean pre-intervention outcome levels in England. Authors' analysis of Worldpanel by Numerator's OOH Purchase panel, 47w/e, 27th Nov 2022.

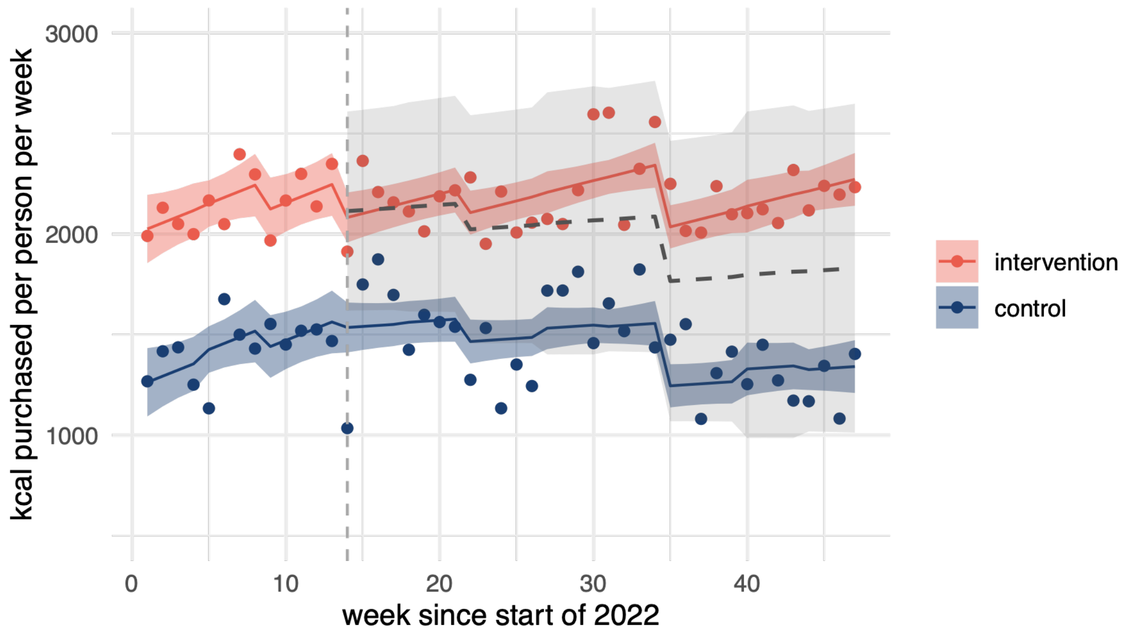

Fig S1. Overall calories purchased out of home by individuals with overweight. Graph shows observed (points) and predicted (solid lines) calories with counterfactual (grey dashed line) and 95% confidence intervals (ribbons). Implementation of mandatory calorie labelling = week 14. The intervention series includes all out-of-home food and drink purchases in England. The control series is constructed from purchases made in Scotland and Wales and excludes purchases from large chains. Large chains denote restaurant and takeaway businesses identified in the data that have ≥250 employees. Authors' analysis of Worldpanel by Numerator's OOH Purchase panel, 47w/e, 27th Nov 2022.
