# Supplementary material for: Changes in out-of-home food purchasing following the introduction of England’s calorie labelling regulations: a population-level controlled interrupted time series analysis
Source: BMJ Public Health. 2026 Apr 27;4(2):e003957. doi: 10.1136/bmjph-2025-003957 (PMC13141202; doi:10.1136/bmjph-2025-003957)
Supplement: online supplemental file 5 [file bmjph-4-2-s005.pdf]

Changes in out-of-home food purchasing following the introduction of England's calorie labelling regulations: a population-level controlled interrupted time series analysis

Supplementary Material 5 – Robustness checks

Robustness checks

This document contains results from the robustness checks of 'Changes in out-of-home food purchasing following England's calorie labelling regulations: a population-level controlled interrupted time series'.

Table of Contents

1 INCLUDING VARYING PRE-INTERVENTION TRENDS 3

2 PURCHASES MADE FOR THE INDIVIDUAL THEMSELVES ONLY 4

3 PURCHASES MADE BY INDIVIDUALS ≥ 18 YEARS 5

4 EXCLUDING TOP 1% & 10% HIGH-CALORIE PURCHASE OCCASIONS 6

5 BALANCED OBSERVATIONS 8

6 TEMPORAL FALSIFICATION 9

7 INCLUDING A PRICE INDEX 10

1 Including varying pre-intervention trends

In this robustness check, we included an interaction term between pre-intervention time and group (intervention/control), thereby letting time trends vary by group.

Table S4. Immediate (level) and longer-term (slope) effects of mandatory calorie labelling at population level – sensitivity analysis 1: including varying pre-intervention trends

Outcome (kcal per person per week)	Level change (95%CI)	P	Slope change (95%CI)	P
All calories purchased OOH	-67.8 (-477.01 to 341.5)	0.743	-1.3 (-35.9 to 33.4)	0.943
From large chains [a]	-80.5 (-428.8 to 267.8)	0.647	20.6 (-8.9 to 50.1)	0.169
From non-chains	-163.8 (-498.8 to 171.2)	0.338	6.3 (-22.1 to 34.6)	0.666
From meals	-76.2 (-415.9 to 263.5)	0.656	7.7 (-21.0 to 36.5)	0.595
From higher-calorie coffees	-3.8 (-27.4 to 19.7)	0.747	-0.14 (-2.1 to 1.7)	0.892
From lower-calorie coffees	1.5 (-2.8 to 5.8)	0.481	0.21 (-0.15 to 0.58)	0.246
From sandwiches	-47.3 (-128.2 to 33.5)	0.247	1.6 (-5.2 to 8.5)	0.637
From fish and chip meals	27.6 (-44.4 to 99.5)	0.448	-3.2 (-9.3 to 2.9)	0.305

95%CI = 95% confidence interval; OOH = out of home. [a] Large chains denote restaurant and takeaway businesses identified in the data that have ≥250 employees. Authors' analysis of Worldpanel by Numerator's OOH Purchase panel, 47w/e, 27th Nov 2022.

2 Purchases made for the individual themselves only

This robustness check considers only purchases reported by the individual as purchased for themselves only (57.8% of all transactions). The main analysis also includes purchases made by the individual for other adults and children.

Table S5. Immediate (level) and longer-term (slope) effects of mandatory calorie labelling at population level – sensitivity analysis 2: purchases made for the individual themselves only

Outcome (kcal per person per week)	Level change (95%CI)	P	Slope change (95%CI)	P
All calories purchased OOH	-105.8 (-278.6 to 66.9)	0.230	-1.2 (-6.1 to 3.8)	0.647
From large chains [a]	-51.5 (-210.4 to 107.4)	0.525	2.0 (-2.5 to 6.5)	0.387
From non-chains	-116.2 (-279.6 to 47.3)	0.164	-1.3 (-6.0 to 3.3)	0.575
From meals	-70.5 (-201.9 to 61.0)	0.294	**3.7 (0.01 to 7.5)**	**0.050**
From higher-calorie coffees	-4.5 (-19.2 to 10.1)	0.544	**0.47 (0.06 to 0.89)**	**0.026**
From lower-calorie coffees	1.9 (-1.5 to 5.3)	0.279	**0.27 (0.17 to 0.36)**	**<0.001**
From sandwiches [b]	-22.4 (-77.1 to 32.3)	0.423	**-2.5 (-4.3 to -0.64)**	**0.008**
From fish and chip meals	1.9 (-36.7 to 40.4)	0.925	-0.19 (-1.3 to 0.92)	0.740

95%CI = 95% confidence interval; OOH = out of home. [a] Large chains denote restaurant and takeaway businesses identified in the data that have ≥250 employees. [b] Autocorrelation detected and robust standard errors used. Authors' analysis of Worldpanel by Numerator's OOH Purchase panel, 47w/e, 27th Nov 2022.

3 Purchases made by individuals ≥ 18 years

This robustness check considers only purchases reported by individuals 18 years or above (98.7% of all transactions).

Table S6. Immediate (level) and longer-term (slope) effects of mandatory calorie labelling at population level – sensitivity analysis 3: purchases made for the individuals ≥18 years

Outcome (kcal per person per week)	Level change (95%CI)	P	Slope change (95%CI)	P
All calories purchased OOH	-69.4 (-445.9 to 307.2)	0.718	-0.39 (-32.4 to 31.6)	0.981
From large chains [a]	-77.4 (-400.0 to 245.1)	0.638	20.7 (-6.7 to 48.0)	0.138
From non-chains	-125.7 (-422.5 to 171.2)	0.407	6.5 (-1.9to 14.9)	0.132
From meals	-88.6 (-403.1 to 225.9)	0.581	8.9 (-17.7 to 35.6)	0.512
From higher-calorie coffees	-2.3 (-25.7 to 21.2)	0.851	-0.15 (-2.14 to 1.84)	0.883
From lower-calorie coffees	1.5 (-2.8 to 5.7)	0.503	0.23 (-0.13 to 0.59)	0.209
From sandwiches	-40.6 (-117.3 to 36.1)	0.300	1.2 (-5.3 to 7.7)	0.708
From fish and chip meals	28.5 (-42.0 to 99.0)	0.429	-2.9 (-8.8 to 3.1)	0.347

95%CI = 95% confidence interval; OOH = out of home. [a] Large chains denote restaurant and takeaway businesses identified in the data that have ≥250 employees. Authors' analysis of Worldpanel by Numerator's OOH Purchase panel, 47w/e, 27th Nov 2022.

4 Excluding top 1% & 10% high-calorie purchase occasions

In this robustness check we removed purchases made during the purchase occasions with the top 1% / top 10% highest calorie count of items purchased during the occasion. This allower-calories us to assess the impact of these outlier purchases on the estimated intervention effect.

Table S7. Immediate (level) and longer-term (slope) effects of mandatory calorie labelling at population level – sensitivity analysis 4a: excluding top 1% of high-calorie purchase occasions

Outcome (kcal per person per week)	Level change (95%CI)	P	Slope change (95%CI)	P
All calories purchased OOH	-110.8 (-409.2 to 187.5)	0.467	1.5 (-7.0 to 9.8)	0.734
From large chains	-49.0 (-318.8 to 220.8)	0.722	5.4 (-2.2 to 13.1)	0.165
From non-chains [a]	-143.7 (-408.7 to 121.2)	0.288	1.0 (-6.5 to 8.5)	0.792
From meals	-91.2 (-327.4 to 144.9)	0.449	4.8 (-2.0 to 11.5)	0.165
From higher-calorie coffees	-5.7 (-27.2 to 15.8)	0.602	0.47 (-0.14 to 1.1)	0.130
From lower-calorie coffees	1.1 (-2.8 to 5.0)	0.592	**0.30 (0.19 to 0.41)**	**<0.001**
From sandwiches	-30.7 (-105.6 to 44.2)	0.422	-1.9 (-4.0 to 0.22)	0.078
From fish and chip meals	-15.4 (-66.2 to 35.5)	0.554	-0.7 (-2.2 to 0.7)	0.329

95%CI = 95% confidence interval; OOH = out of home. [a] Large chains denote restaurant and takeaway businesses identified in the data that have ≥250 employees. Authors' analysis of Worldpanel by Numerator's OOH Purchase panel, 47w/e, 27th Nov 2022.

Table S8. Immediate (level) and longer-term (slope) effects of mandatory calorie labelling at population level – sensitivity analysis 4b: excluding top 10% of high-calorie purchase occasions

Outcome (kcal per person per week)	Level change (95%CI)	P	Slope change (95%CI)	P
All calories purchased OOH	-119.1 (-272.3 to 34.1)	0.128	-2.8 (-7.1 to 1.6)	0.212
From large chains [a]	-91.1 (-228.6 to 46.5)	0.194	0.11 (-3.7 to 3.9)	0.956
From non-chains	-118.8 (-263.0 to 25.4)	0.106	-2.9 (-6.9 to 1.2)	0.173
From meals	**-97.0 (-188.8 to -5.2)**	**0.038**	0.8 (-1.8 to 3.4)	0.560
From higher-calorie coffees	-5.7 (-26.1 to 14.6)	0.580	0.47 (-0.10 to 1.1)	0.107
From lower-calorie coffees	2.4 (-1.1 to 5.8)	0.178	**0.30 (0.21 to 0.40)**	**<0.001**
From sandwiches	-28.8 (-102.4 to 44.8)	0.443	-2.0 (-4.1 to 0.05)	0.055
From fish and chip meals	-4.8 (-27.3 to 17.7)	0.673	**-1.3 (-2.0 to -0.57)**	**<0.001**

95%CI = 95% confidence interval; OOH = out of home. [a] Large chains denote restaurant and takeaway businesses identified in the data that have ≥250 employees. Authors' analysis of Worldpanel by Numerator's OOH Purchase panel, 47w/e, 27th Nov 2022.

5 Balanced observations

In this robustness check, we restrict the post-intervention series to 13 weeks to match the pre-intervention series.

Table S9. Immediate (level) and longer-term (slope) effects of mandatory calorie labelling at population level – sensitivity analysis 5: using balanced observations pre and post labelling

Outcome (kcal per person per week)	Level change (95%CI)	P	Slope change (95%CI)	P
All calories purchased OOH [a]	-123.1 (-490.9 to 244.7)	0.512	-8.1 (-34.9 to 18.6)	0.552
From large chains [b]	11.2 (-367.0 to 389.3)	0.954	4.9 (-17.3 to 27.0)	0.667
From non-chains	-107.5 (-478.4 to 263.5)	0.570	-7.1 (-28.9 to 14.6)	0.519
From meals	-61.0 (-421.5 to 299.6)	0.740	-4.6 (-25.7 to 16.5)	0.669
From higher-calorie coffees	-5.7 (-36.4 to 25.1)	0.718	-0.4 (-2.2 to 1.4)	0.645
From lower-calorie coffees	-0.49 (-5.7 to 4.7)	0.855	0.21 (-0.09 to 0.52)	0.175
From sandwiches	4.7 (-73.8 to 83.3)	0.906	-3.4 (-8.0 to 1.2)	0.147
From fish and chip meals	10.1 (-76.4 to 96.5)	0.820	-1.9 (-7.0 to 3.1)	0.459

95%CI = 95% confidence interval; OOH = out of home. [a] Autocorrelation detected and robust standard errors used. [b] Large chains denote restaurant and takeaway businesses identified in the data that have ≥250 employees. Authors' analysis of Worldpanel by Numerator's OOH Purchase panel, 47w/e, 27th Nov 2022.

6 Temporal falsification

In this robustness check, we moved the intervention 'backwards' by four weeks to the week commencing 7th March 2022.

Table S10. Immediate (level) and longer-term (slope) effects of mandatory calorie labelling at population level – sensitivity analysis 6: temporal falsification (intervention 4 weeks earlier)

Outcome (kcal per person per week)	Level change (95%CI)	P	Slope change (95%CI)	P
All calories purchased OOH	0.24 (-267.5 to 268.0)	0.999	6.9 (-4.1 to 17.8)	0.218
From large chains [a]	-70.4 (-299.1 to 158.3)	0.547	8.7 (-0.63 to 18.0)	0.068
From non-chains	29.9 (-192.8 to 252.6)	0.793	7.4 (-1.7 to 16.5)	0.109
From meals	-58.8 (-280.8 to 163.2)	0.604	8.6 (-0.49 to 17.6)	0.064
From higher-calorie coffees	0.54 (-14.8 to 15.9)	0.945	0.59 (-0.03 to 1.2)	0.063
From lower-calorie coffees	-0.19 (-3.1 to 2.7)	0.895	**0.29 (0.18 to 0.41)**	**<0.001**
From sandwiches	-3.9 (-57.0 to 49.1)	0.884	-1.7 (-3.8 to 0.49)	0.128
From fish and chip meals	-17.2 (-63.9 to 29.5)	0.470	-0.43 (-2.3 to 1.5)	0.655

95%CI = 95% confidence interval; OOH = out of home. [a] Large chains denote restaurant and takeaway businesses identified in the data that have ≥250 employees. Authors' analysis of Worldpanel by Numerator's OOH Purchase panel, 47w/e, 27th Nov 2022.

7 Including a price index

This robustness check includes indices for prices of food and drink items purchased out of home created from the purchase data. This index was expressed as average spend per 100 kcal across all items purchased in each week and region, and does not account for sales.

Table S11. Immediate (level) and longer-term (slope) effects of mandatory calorie labelling at population level – sensitivity analysis 7: including a price index of average price per item

Outcome (kcal per person per week)	Level change (95%CI)	P	Slope change (95%CI)	P
All calories purchased OOH	-76.3 (-453.4 to 300.7)	0.692	6.26 (-4.6 to 17.1)	0.258
From large chains [a]	-13.7 (-335.2 to 307.8)	0.934	**10.3 (1.0 to 19.5)**	**0.030**
From non-chains	-135.4 (-447.4 to 176.5)	0.395	5.4 (-3.6 to 14.3)	0.242
From meals	-70.5 (-384.8 to 243.7)	0.660	8.7 (-0.3 to 17.8)	0.058
From higher-calorie coffees	-7.6 (-29.2 to 14.1)	0.495	0.37 (-0.26 to 0.99)	0.247
From lower-calorie coffees	1.2 (-2.8 to 5.2)	0.556	**0.30 (0.19 to 0.41)**	**<0.001**
From sandwiches	-22.9 (-95.6 to 49.8)	0.537	-1.5 (-3.5 to 0.65)	0.175
From fish and chip meals	12.1 (-55.1 to 79.2)	0.725	0.06 (-1.9 to 2.0)	0.949

95%CI = 95% confidence interval; OOH = out of home. [a] Large chains denote restaurant and takeaway businesses identified in the data that have ≥250 employees. Authors' analysis of Worldpanel by Numerator's OOH Purchase panel, 47w/e, 27th Nov 2022.
